# A Portion of a Tooth Imbedded in Lower Lip

**Published:** 1876-05

**Authors:** Francis Mason

**Affiliations:** before the Odontological Society of Great Britain.


### SELECTED ARTICLES.

---

### ARTICLE IV.

### *Remarks on a Case in Which a Portion of a Tooth was Embedded in the Lower Lip.*

By Francis Mason, F. R. C. S., before the Odontological Society of Great Britain.

It often happens that a case of interest will be well-nigh forgotten until another presents itself which prompts us to refer to the first one. In bringing the following case before the members of the Society, I am the more induced to do so because, in looking through the medical periodicals, I have chanced to find a few other examples closely resembling the one I have the honor of submitting to the meeting. The case is this: A patient, aged twenty-two, applied to me in 1868 at the Westminister Hospital with a swelling of the lower lip on the left side. He had been told he had cancer, and sought advice on that account. It appeared that he had fallen from a scaffolding two months previously and had sustained considerable injury to his scalp and face, and his lower jaw was fractured. He had been a patient elsewhere, and stated that when he was under treatment he had fits. On examining the part the lip was found a good deal swollen, and rather tender to the touch. I made a puncture, when the knife impinged on a hard substance, which proved to be a portion of the crown of a life lateral incisor of the lower jaw, which the patient said he missed after the accident. It was decayed at the neck, and had sharp edges. The patient recovered, and was only under observation for a few days afterwards. I say I have referred to the medical journals and have found other cases; and with your permission, as they are not long, I will read the particulars of them. In the *Lancet* (May 17, 1862) reference is made to a case in which a canine tooth was found lodged in the thickness of the lower lip, simulating a cancerous tumor. "The patient was a lady, forty years, of age, who had always suffered with her teeth, and had but few incisors left, the rest of the jaws presenting roots more or less firmly wedged, and the alveoli more or less decayed. Towards the end of the year 1854 she felt a small tumor forming in the lower lip of the left side, which tumor soon filled the space below the internal aspect of the lip and gum. Pain was subsequently experienced, and a few months afterwards the patient could hardly eat. She sent for M. Landyck, who discovered a large tumor situated as above, and ulcerating. Ablation was not proposed, as the lady was very nervous, and cauterization was resorted to. Whilst using the caustic some time after, M. Landyck felt a hard substance in the tumor. By then making a crucial incision, he discovered a long root of the canine tooth, covered with a thick layer of calcareous matter. It was placed horizontally, the apex turned towards the lip, and its upper part adhering to the bone. This root being removed, the tumor disappeared, and the patient was freed from all uneasiness." Another case is related in the *Lancet* (October, 1868.) The patient was under the care of Mr. W. H. Folker, at the North Staffordshire Infirmary, who has favored me with a note respecting the name. His report is this: The patient was sixteen years of age, a joiner, and was admitted August 29, 1867, on account of a tumor existing in the substance of the upper lip. The swelling was on the right of the mesial line, corresponding to the right central incisor tooth (which was noticed to be wanting,) and it seemed to be formed by hypertrophied lip. It caused a good deal of deformity, as the patient was unable accurately to close his lips. The tumor felt hard, but was not painful. On carefully examining the swelling a small aperture was perceived at its base; and on passing a fine probe a hard substance was felt, which was diagnosed to be a tooth. On August 31st, an incision having been made through the tumor, a tooth was found at its base. On attempting to extract it by the forceps it was fractured; this proved to be owing to its peculiar shape, being of a crescentic form, and passing almost at right angles to the alvolus. After its removal, the swelling disappeared. and the patient was discharged on the following day. Then there is another case which I think of interest. It is one of migration of a tooth; and appeared in the *Medical Times* (April 30ih, 1864,) from the "Union Medicale," No. 142. "M. Delestre related to the Paris Medical Society the case of a lady upon whose palate, opposite the front large molar (which was missing,) and about a centimetre from the edge of the gum, was observed a small, rounded, blackened loss of substance which seemed as if it had been punched out. Various surgeons who had seen it had regarded it as caries of the maxillary bone, and treatment had been employed in vain. M. Delestre found on examination with a probe, that there was not the crepitation produced by contact with caries. and that a certain amount of mobility existed. Passing in a delicate forceps, to his great surprise he withdrew the fang of a tooth, about a centimetre in size, which, proceeding from the first molar, had evidently gone through the substance of the maxillary bone and become placed perpendicularly to the palate. The patient remained entirely well." Mr. Haynes Walton has reported a case in the *Medical Times* (November, 1869,) of "a patient, aged thirty-five, who, three years and a half before, had a fall, and lost his right upper lateral incisor. He had much pain and a few weeks later an abscess formed, which discharged, through a small opening in the cheek, as well as through the alveolus of a lost tooth. Then another abscess formed, leaving a sinus open in the right cheek. On probing this a hard substance was found. The wound opened up, and a perfect incisor tooth lying loose in the antrum was removed." I think, sir, the above cases are interesting on account of their rarity, but I bring them before the Society for another reason, namely, that a misplaced tooth, acting as a foreign body, may occasionally be the cause of very severe constitutional disorder. In the first case mentioned it will be remembered there was a history of the patient having had fits. Every member here knows that carious teeth give rise to all sorts af ailments—neuralgia, deafness, blindness, amaurosis, squint, wryneck, closure of jaws, epilepsy, &c., and in connection with these cases I venture to direct the attention of the Society to a paper read by Dr. George Johnson, at the Clinical Society, on November 8th, 1872, showing what small matters will give rise to serious nervous disturbance. The case he speaks of was one in which tetanus with facial neuralgia and palsy, and a recurrence of epilepsy, were excited by a foreign body in a wound on the cheek, The patient was a wheelwright, aged forty four, who, on July 4th, 1872, was cut on the cheek by a blow from an iron axle that fell upon his face. The wound was strapped by a chemist, and healed, but remained very painful. On the 12th he had an epileptic fit. In early life he had been subject to epilepsy, but, until the occasion mentioned, had been free from fits for twelve years. On the morning of the 13th he had difficulty in opening his mouth. The left masseter muscle felt permanently hard and rigid. There was a scar about three-fourths of an inch long an inch below the left eye, the cicatrix being hard and very tender to the touch. Dr. Johnson believing that there was a foreign body present, directed the house surgeon to cut through the cicatrix, when a sharp, angular piece of flint, nearly as large as a grain of wheat, was discovered and removed. With this the symptoms gradually passed away. Dr. Johnston also related another case of a similar kind. A boy, aged thirteen, was admitted for tetanic rigidity of the trunk and extremities. The symptoms, which had lasted more than a week, had commenced about ten days after a wound on the thigh by a sharp piece of wood. The wound had healed, but the cicatrix was hard and tender. The house surgeon was requested to incise the cicataix, when a piece of woolen material about the size of a small pea was discovered and removed. Dr Johnson remarked that the chief interest of the two cases consisted in the fact that formidable nervous symptoms were excited by the presence of a foreign body of small size beneath the cicatrix of a recently healed wound. In conclusion, I may refer to a case which was under my care at the Westminster Hospital, when I was surgeon there, of a girl who was struck on the head by a rolling pin, and she was admitted in consequence of having tetanic symptoms. She died of tetanus, and, after death, we found, on removing the scalp, that there was a piece of hair-pin, nearly an inch in length, which was, no doubt, the cause of the tetanus. The case is additionally interesting, because a portion of the rectus abdominis muscle had been ruptured by a tetanic spasm. One other case of some interest I may mention. It is quoted in the "British Journal of Dental Science," (vol. 5,) and relates to a molar tooth found lodged in the tongue. "At the battle of Williamsburg," says the report, "a man received a bullet-wound in the lower jaw, which was shattered. After attendance at the hospital, however, the wound healed rapidly. Some weeks afterwards he again presented himself, and on examination it was discovered that a molar tooth which had been knocked out of the jaw had been buried in the tongue." I have nothing to add to the examples that you have permitted me to bring before the Society, and in thanking you for your courteous attention, beg to ask, for my own information, if you, sir, or any members present, have met with cases in which pieces of teeth have remained for any length of time embedded in the lips, or in the cheek, or in any other part? I venture to think that we may learn one lesson, at least, from the instances I have quoted, and it is to look carefully for any foreign body, however small, in patients who have nervous symptoms of an anomalous character.—*Dental Miscellany.*

